# Non-replicating vaccinia virus NTV as an effective next-generation smallpox and monkeypox vaccine: evidence from mouse and rhesus monkey models

**DOI:** 10.1080/22221751.2023.2278900

**Published:** 2023-12-06

**Authors:** Qiaohong Chu, Baoying Huang, Mengzhe Li, Xueting Cheng, Shuting Huo, Jiao Ren, Yao Deng, Wenjie Tan

**Affiliations:** a NHC Key Laboratory of Biosafety, National Institute for Viral Disease Control and Prevention, Chinese Center for Disease Control and Prevention, Beijing, People’s Republic of China; b School of Public Health, Xinxiang Medical University, Xinxiang, People’s Republic of China

The mpox (formerly known as monkeypox) outbreak in May 2022 drew considerable attention towards this disease [1]. Smallpox virus, vaccinia virus (VACV), and monkeypox virus (MPXV) belong to the orthopoxvirus genus, accordingly, based on real-world data, VACV vaccination constitutes an effective tool against smallpox and mpox [2,3]. Currently, both ACAM2000 (a second-generation vaccine against smallpox) and MVA-BN (a live, non-replicating modified vaccinia Ankara vaccine, also known as JYNNEOS) have been approved for use in humans against mpox [4,5]. The vaccinia virus Tiantan (VTT) strain has been widely used as a smallpox vaccine in China. After eradicating smallpox in China in 1979, vaccination with VTT for the general population was discontinued in 1980. Accordingly, individuals born before 1980 were highly likely to be vaccinated, whereas those born after 1980 were not vaccinated with VTT. The non-replicating VACV Tian Tan (NTV), which is also derived from the Tian Tan strain, has a better safety profile than VTT [6]. As no data on cross-neutralizing antibodies against mpox among VTT vaccinee are currently available, it is unclear whether NTV has a similar immunoprotective potential as that of VTT against mpox. This study primarily aimed to evaluate the postvaccination neutralizing antibody titres against the mpox virus (after either VTT or NTV inoculation) in mice, rhesus monkeys, and humans. The titres of the samples were determined using plaque-reduction neutralization tests (PRNTs). We found that although VTT vaccination confers cross-humoral immunity against mpox in humans, residual immunity with these vaccinee remains relatively low. Furthermore, NTV and VTT had similar efficacy in inducing cross-protective immunity against mpox in mouse and rhesus monkey models.

Following smallpox eradication in 1980, mpox became the most important transmissible orthopoxvirus-related disease worldwide. The current mpox outbreak is primarily affecting men who have sex with men, particularly those who have multiple sexual partners, and vaccination is a crucial preventive measure. Data on the efficacy of VTT smallpox vaccines against MPXV in humans are scarce. Despite immunosenescence in vaccinated individuals, smallpox vaccination elicits long-lasting immunity against smallpox [7,8]. A study conducted in Italy showed that antibodies evoked by first-generation smallpox vaccines could neutralize MPXV Clade II infections for more than 40 years after vaccination [3]. First, we aimed to determine whether individuals vaccinated with VTT could produce cross-reactive antibodies against MPXV. In this study, serum samples were collected from 30 individuals. The participants were categorized into two groups based on their year of birth (i.e. before or after 1980; Table S1), which corresponded to the cessation of smallpox vaccination in 1980. PRNTs demonstrated substantial differences in serum positivity rates for antibodies against vaccinia and MPXV among various age groups in China (Figure 1(A)). Individuals born after 1980 exhibited a seroprevalence of 0 (0/13) for neutralizing antibodies (nAbs) against VTT and MPXV, whereas those born before 1980 showed a seroprevalence of 70.6% (12/17) and 58.8% (10/17) for nAbs against VTT and MPXV, respectively. Individuals born before 1980 had higher geometric mean titres (GMTs) of serum nAbs against VTT and MPXV, respectively, than those born after 1980. Unfortunately, we cannot obtain specific information on VTT vaccination for individuals born before 1980 because of its long history and personal privacy. It is uncertain whether the absence of antibodies against VTT and MPXV for individuals enrolled in this study born before 1980 is related to the waning of antibodies over time. The relatively low-level residual nAb immunity against the mpox virus in the Chinese population suggests the need for consistent and rigorous public health interventions.

**Figure 1.**
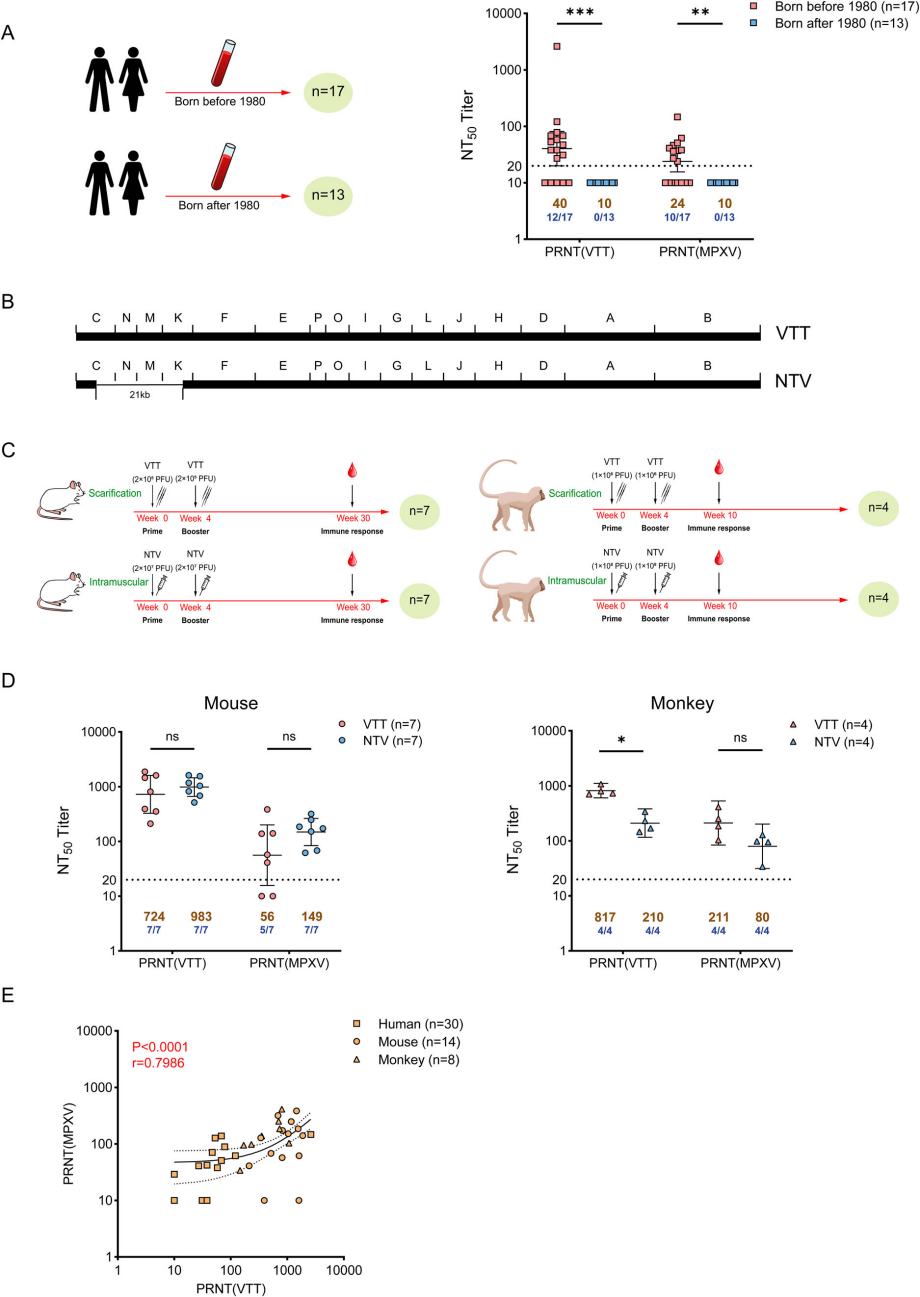
Neutralization activities against both VTT and MPXV in mice,monkey and human induced by vaccination with either VTT or NTV. (A) Experimental design and nAbs titre (shown as NT50) against VTT or MPXV detected by PRNT in blood donors of human. The individual information of blood donors in this study is shown in supplemental Table 1. (B) Schematic diagram of VTT and NTV genome. (C) Experimental design of VTT and NTV vaccination in mice (left) and monkey (right). (D) Neutralization activities (shown as NT50) against VTT or MPXV detected by PRNT in mice (left) or monkey (right). (E) Correlation between NT50 of VTT and NT50 of MPXV based on PRNT. A regression model was fitted to log-transformed titre to give r and *P* values as indicated. Model prediction is shown with the 95% confidence interval (indicating as dotted line connecting points).

Compared with that of the original virus strain, the genome of NTV lost 21,243 nucleosides following the deletion of 26 genes associated with host range and virulence between the C and K digestion fragments of HindIII (Figure 1(B)). Although it lacks replicative ability in primates and humans, NTV can propagate well in primary chick embryo fibroblasts, is markedly safer than VTT, and can be administered intramuscularly. As VTT with intradermal inoculation, which can achieve a better immune response than intramuscular injection, had been used for vaccination against smallpox and mpox. NTV has defective in vivo replication for human, and the intramuscular injection can be used as a more safe and convenient way for immunization, but a higher dose is required for intramuscular immunization with NTV. Our previous study showed that robust humoral and cellular immunity could be induced in mice and monkeys via intramuscular injection with recombinant NTV [6,9]. Our study aimed to investigate whether animals immunized with VTT and NTV could generate cross-reactive antibodies against MPXV.

To assess the efficacy of NTV and VTT in inducing humoral immune responses against VACV and MPXV infections in mice and monkeys, we categorized the animals into the following two groups: one group of mice (*n* = 7) was inoculated with 2 × 10^5^ PFU of replicating VACV VTT via skin scratches, whereas the other group (*n* = 7) received 2 × 10^7^ PFU of NTV via intramuscular injection. Similarly, one group of monkeys (*n* = 4) was inoculated with 10^6^ PFU VTT via skin scratches, whereas the other group (*n* = 4) received 10^8^ PFU NTV via intramuscular injection. The specific immunization regimen is shown in Figure 1(C). The mice and monkeys were euthanised after the administration of the second dose, and serum was collected for further analysis. The titres of nAbs against VTT and MPXV in the serum samples were determined using a plaque-reduction neutralization assay. The results revealed that NTV induced cross-neutralization of MPXV in both the mouse and rhesus monkey models (Figure 1(D)). In mice, intramuscular injection of NTV induced similar levels of nAbs against VACV and cross-neutralization against MPXV as that obtained with the skin-scratch inoculation of VTT. The GMT of nAbs against NTV-induced VTT was 1.4-fold higher than that induced by VTT. Similarly, the GMT of serum nAbs against MPXV induced by the NTV was 2.7-fold higher than that induced by VTT. In monkeys, intramuscular injection of NTV induced weaker neutralization of VACV and cross-neutralization of MPXV than that achieved via the skin-scratch inoculation of VTT. The GMT of serum nAbs against VTT that were induced by VTT vaccination was 3.9-fold higher than that induced by NTV. Moreover, the GMT of serum nAbs against MPXV that were induced by VTT vaccination was 2.6-fold higher than that of nAbs induced by NTV. This unexpected disparity in monkey nAbs against VACV between the VTT and NTV warrants further investigation.

Finally, regression analysis was conducted to ascertain the correlation between the GMT of nAbs against MPXV and VACV based on the PRNTs in this study, and we observed good consistency (Figure 1(E)). PRNTs based on VACV, which can be performed in a BSL-2 facility, might constitute an alternative assay for detecting nAbs against MPXV.

This study had certain limitations. First, although we assessed the vaccination-induced humoral immune response, future studies should examine the cellular immune response, which is a vital aspect of vaccine efficacy. Second, it is essential to establish whether NTV can elicit sustained immunity comparable to that of traditional VTT, as shown in Figure 1 and Table S1. Furthermore, the sample size of the population and monkeys included in this study was small because of time constraints and ethical restrictions; therefore, we recommend the collection of additional serum samples from individuals who were previously vaccinated with VTT to analyse the relationship between residual immunity and age. However, our population study's results are consistent with those reported in other studies [3,7,8].

In summary, given the low level of residual immunity against VACV and MPXV in the Chinese population, further development of a potential vaccine against mpox is crucial to address the current public health crisis. More importantly, the results presented here show that vaccination with the non-replicating VACV NTV is as effective as that with the replicating VACV VTT in stimulating nAbs against mpox and VACV in mouse and rhesus monkey models. Based on experimental evidence, we suggested that the NTV was a better choice as a next-generation, safer smallpox and monkeypox vaccine, similar to MVA-BN, which has fewer side effects than the first-generation vaccine and the second-generation vaccine (VTT, ACAM2000).

## Supplementary Material

SUPPLEMENT_MATERIALS_AND_METHODS_EMI_NTV

## References

[1] Venkatesan P. Global monkeypox outbreak. Lancet Infect Dis. 2022;22(7):950. doi:10.1016/S1473-3099(22)00379-6 35752185 PMC9533939

[2] Edghill-Smith Y, Golding H, Manischewitz J, et al. Smallpox vaccine–induced antibodies are necessary and sufficient for protection against monkeypox virus. Nat Med. 2005;11(7):740-747. doi:10.1038/nm1261 15951823

[3] Criscuolo E, Giuliani B, Ferrarese R, et al. Smallpox vaccination-elicited antibodies cross-neutralize 2022-monkeypox virus clade II. J Med Virol. 2023;95(3). doi:10.1002/jmv.28643 36890648

[4] Ilchmann H, Samy N, Reichhardt D, et al. One- and two-dose vaccinations with modified vaccinia Ankara-Bavarian nordic induce durable B-cell memory responses comparable to replicating smallpox vaccines. J Infect Dis. 2023;227(10):1203-1213.doi:10.1093/infdis/jiac455 36408618 PMC10175071

[5] Zaeck LM, Lamers MM, Verstrepen BE, et al. Low levels of monkeypox virus-neutralizing antibodies after MVA-BN vaccination in healthy individuals. Nat Med. 2022;29(1):270-278. doi:10.1038/s41591-022-02090-w 36257333 PMC9873555

[6] Wen B, Deng Y, Chen H, et al. The novel replication-defective vaccinia virus (Tiantan strain)–based hepatitis C virus vaccine induces robust immunity in macaques. Mol Ther. 2013;21(9):1787-1795. doi:10.1038/mt.2013.122 23774793 PMC3776631

[7] Chan CEZ, Wong SKK, Yazid NBM, et al. Residual humoral immunity sustained over decades in a cohort of vaccinia-vaccinated individuals. J Infect Dis. 2023;227(8):1002-1006. doi:10.1093/infdis/jiac409 36200239

[8] Pütz MM, Alberini I, Midgley CM, et al. Prevalence of antibodies to vaccinia virus after smallpox vaccination in Italy. J Gen Virol. 2005;86(11):2955–2960. doi:10.1099/vir.0.81265-0 16227216

[9] Zhan Y, Deng Y, Huang B, et al. Humoral and cellular immunity against both ZIKV and poxvirus is elicited by a two-dose regimen using DNA and non-replicating vaccinia virus-based vaccine candidates. Vaccine. 2019;37(15):2122–2130. doi:10.1016/j.vaccine.2019.02.063 30851967

