# Supplementary material for: Non-replicating vaccinia virus NTV as an effective next-generation smallpox and monkeypox vaccine: evidence from mouse and rhesus monkey models
Source: Emerg Microbes Infect. 2023 Dec 6;12(2):2278900. doi: 10.1080/22221751.2023.2278900 (PMC10768732; doi:10.1080/22221751.2023.2278900)
Supplement: SUPPLEMENT_MATERIALS_AND_METHODS_EMI_NTV [file TEMI_A_2278900_SM7132.docx]

# SUPPLEMENT MATERIALS AND METHODS

# Serum samples

All animal experiments were approved by the Committee on the Ethics of Animal Experiments of the Chinese Centre for Disease Control and Prevention (China CDC), and followed the recommendations in the ARRIVE guidelines. The animal immunisation protocols and serum sample sources are summarised in Fig. 1C. A group of BALB/c mice (n=7) underwent primary immunisation by skin scratch vaccination at a dose of 2×10^5^ PFU VTT, followed by a booster with the same dose at 4-week intervals. Another group of BALB/c mice (n=7) received primary immunisation via intramuscular injection at a dose of 2×10^7^ PFU of NTV, and the booster was administered at the same dose at 4-week intervals. Serum samples were collected at 26 weeks after the second dose. Similarly, a group of rhesus macaques (n = 4) received primary immunisation through skin scratch vaccination at a dose of 10^6^ PFU of VTT, and the booster was administered at the same dose at 4-week intervals. An additional group of rhesus macaques (n = 4) received primary immunisation via intramuscular injection at a dose of 10^8^ PFU of NTV, and the booster was administered at the same dose at 4-week intervals. Serum samples were collected 6 weeks after the second dose. Furthermore, 30 human serum samples were collected in China and written informed consent was obtained from all participants (Table S1). The smallpox vaccination and infection histories of the participants were unknown. The following experiments were conducted on the collected serum samples: a plaque-reduction neutralisation test against VTT and a plaque-reduction neutralisation test against MPXV.

# Plaque reduction neutralisation test (PRNT)

The monkeypox virus (clade IIb, B.1.3) and VTT were preserved in our laboratory [9]. Vero cells were seeded into 12-well plates and incubated overnight. Next, 200 µL DMEM (Dulbecco’s Modified Eagle’s Medium) medium with approximately 100–500 PFU virus stocks were mixed with 2-fold serum dilution, followed by the addition of the mixture ((beginning from 1:20 dilution) to Vero cells. The mixture was incubated at 37 °C for 2 h before adding 2 mL DMEM containing 2% FBS (Fetal Bovine Serum) and 1% PS (Penicillin-Streptomycin) per well. After 72 h, the plates were fixed with a cell tissue fixative solution and stained with 0.1% crystal violet. Neutralisation inhibition was calculated based on the number of plaques(Figure S1). The 50% neutralisation titres (NT50s) were calculated using GraphPad Prism 9.5 (GraphPad Software, San Diego, CA, USA) according to the Reed–Muench method. It is important to note that experiments related to the monkeypox virus should only be carried out in a biosafety Level 3 laboratory. NT50 titres against VTT or MPXV in serum based on PRNT among individuals of human, mice and monkey were shown in Table S1,Figure S2 and Figure S3.

# Statistical analysis

All relevant data were analysed using GraphPad Prism 9.5 (GraphPad Software Inc.). Comparisons between two groups were performed by the method of nonparametric Mann-Whitney U-test [10]. Correlation analysis was conducted by the method of Spearman correlation [11]. All hypothesis tests were two-tailed, and the error bars indicate 95% confidence intervals. Statistical significance was determined as follows: * p < 0.05; ** p < 0.01; *** p < 0.001; **** p < 0.0001. Statistical significance was set at P-values less than 0.05.

# REFERENCE

1. Huang B, Zhao H, Song J, et al. Isolation and Characterization of Monkeypox Virus from the First Case of Monkeypox - Chongqing Municipality, China, 2022. China CDC weekly. 2022 Nov 18;4(46):1019-1024. doi: 10.46234/ccdcw2022.206. PubMed PMID: 36483191; PubMed Central PMCID: PMCPMC9713572. eng.

2. Vierra A, Razzaq A, Andreadis A. Chapter 27 - Continuous variable analyses: t-test, Mann–Whitney U, Wilcoxon sign rank. In: Eltorai AEM, Bakal JA, Newell PC, et al., editors. Translational Surgery: Academic Press; 2023. p. 165-170.

3. Faizi N, Alvi Y. Chapter 6 - Correlation∗∗For datasets, please refer to companion site: <https://www.elsevier.com/books-and-journals/book-companion/9780443185502>. In: Faizi N, Alvi Y, editors. Biostatistics Manual for Health Research: Academic Press; 2023. p. 109-126.

**Table S1. Individual information of 30 human sera.**

| **Number** | **Sex** | **Age** | **PRNT50 Against VTT^*^** | **PRNT50 Against MPXV^*^** |
| --- | --- | --- | --- | --- |
| 1 | Male | 2 | -（10） | -（10） |
| 2 | Male | 2 | -（10） | -（10） |
| 3 | Male | 19 | -（10） | -（10） |
| 4 | Female | 22 | -（10） | -（10） |
| 5 | Female | 27 | -（10） | -（10） |
| 6 | Male | 28 | -（10） | -（10） |
| 7 | Female | 30 | -（10） | -（10） |
| 8 | Male | 30 | -（10） | -（10） |
| 9 | Female | 30 | -（10） | -（10） |
| 10 | Female | 33 | -（10） | -（10） |
| 11 | Male | 33 | -（10） | -（10） |
| 12 | Male | 34 | -（10） | -（10） |
| 13 | Male | 38 | -（10） | -（10） |
| 14 | Male | 42 | -（10） | -（10） |
| 15 | Male | 44 | 31 | -（10） |
| 16 | Female | 46 | 2605 | 147 |
| 17 | Male | 47 | 53 | 46 |
| 18 | Female | 48 | -（10） | -（10） |
| 19 | Female | 51 | 78 | 41 |
| 20 | Female | 51 | 121 | 62 |
| 21 | Female | 52 | -（10） | -（10） |
| 22 | Male | 52 | 47 | 37 |
| 23 | Male | 53 | 38 | 24 |
| 24 | Male | 55 | 58 | 38 |
| 25 | Male | 56 | -（10） | -（10） |
| 26 | Female | 57 | 68 | 36 |
| 27 | Male | 57 | 27 | 27 |
| 28 | Female | 60 | -（10） | -（10） |
| 29 | Female | 66 | 68 | 51 |
| 30 | Male | 81 | 38 | -（10） |

Note：^*^We noted that the initial dilution of the tested serum is 1:20 in this study. For the convenience of plotting and statistics in the figure, we assign a value of 10 to the samples tested as negative (indicating as: -).


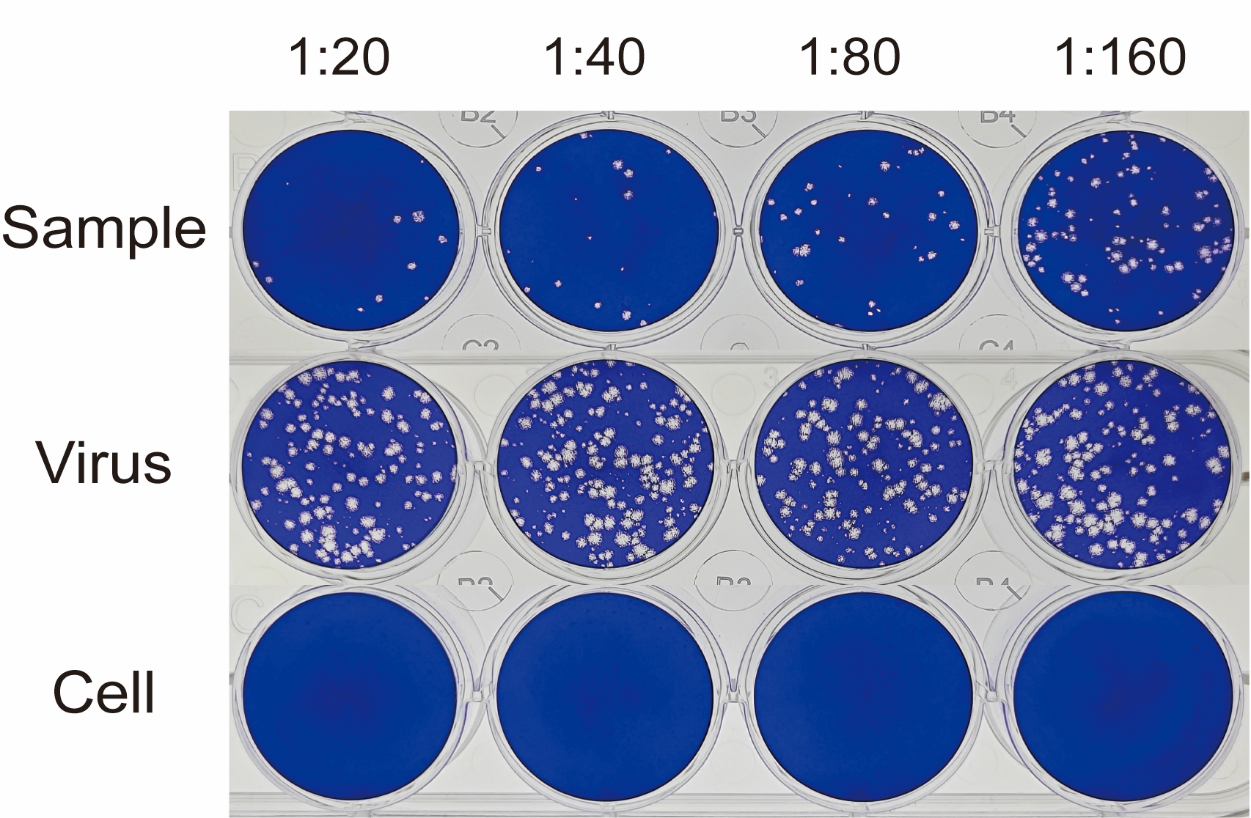


**Figure S1. Representative photos showing MPXV-based PRNT in this study.**


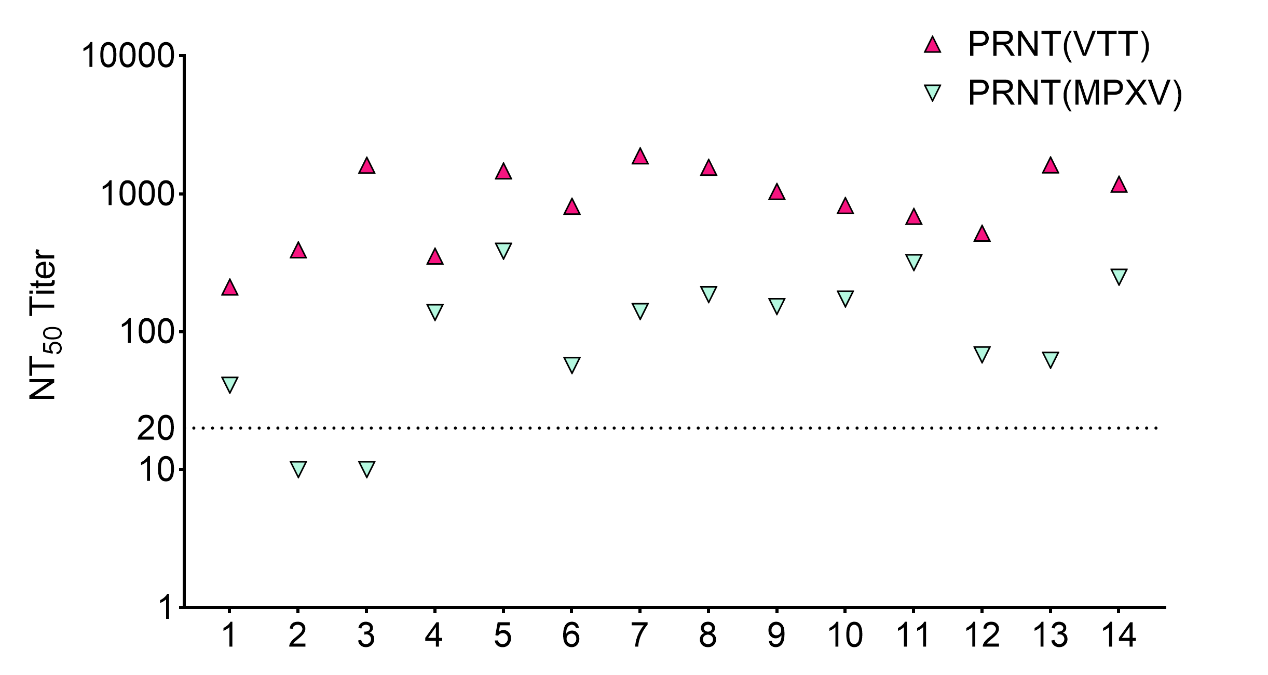
**Figure S2.** **NT50 titres against VTT or MPXV in serum based on PRNT among individuals mice.**


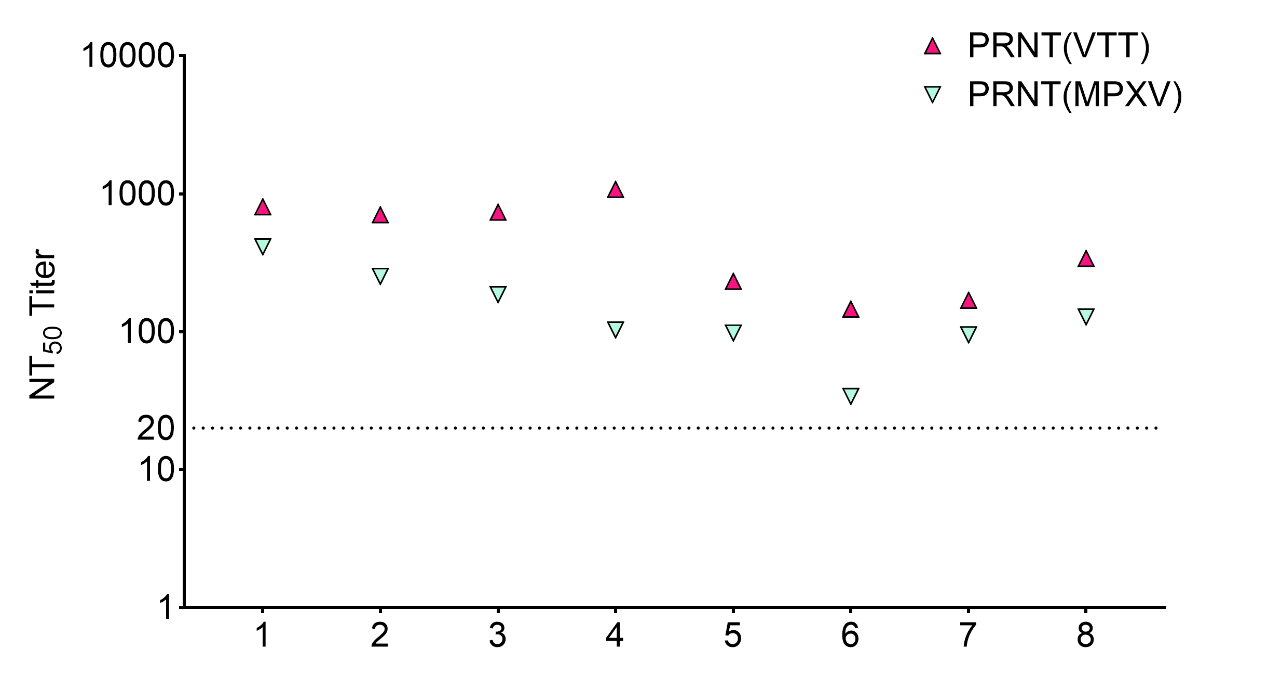
**Figure S3. NT50 titres against VTT or MPXV in serum based on PRNT among individuals monkey.**
